# Dynamic changes in serum IL-6, TNF-*α*, and β₂-microglobulin as early predictors of post-treatment relapse in lymphoma: a prospective cohort study

**DOI:** 10.3389/fmed.2026.1750664

**Published:** 2026-02-12

**Authors:** Yuhao Ma, Jianqiang Song, Chunmeng Song, Xiaolin Zhang

**Affiliations:** 1 Clinical College of Hebei Medical University, Shijiazhuang, Hebei, China; 2 The Second Hospital of Hebei Medical University, Shijiazhuang, Hebei, China; 3 The Third Hospital of Hebei Medical University, Shijiazhuang, Hebei, China

**Keywords:** IL-6, longitudinal biomarkers, lymphoma, minimal residual disease, prospective cohort study, relapse prediction, TNF-*α*, β2-microglobulin

## Abstract

**Background:**

Early identification of relapse after first-line chemotherapy remains a major challenge in lymphoma management. Conventional imaging provides limited sensitivity for minimal residual disease and cannot detect early biochemical changes. This prospective cohort study evaluated whether dynamic changes in serum IL-6, TNF-*α*, and β₂-microglobulin (β₂-MG) can serve as early predictors of relapse.

**Methods:**

A total of 260 patients with pathologically confirmed lymphoma who completed standard chemotherapy were enrolled and followed longitudinally. Serum IL-6, TNF-*α*, and β₂-MG were measured at T1 (end of chemotherapy), T2 (3 months post-treatment), and T3 (6 months post-treatment). Logistic regression, ROC analysis, Kaplan–Meier curves, and Cox proportional hazards models were used to assess predictive performance.

**Results:**

During follow-up, 78 patients (30.0%) experienced relapse. All three biomarkers were significantly elevated in the relapse group at T1, and differences widened at T2 and T3. At T2, IL-6 (adjusted OR = 1.08), β₂-MG (adjusted OR = 1.38), and Ann Arbor stage and IPI score remained independent predictors. β₂-MG exhibited the strongest individual predictive value (AUC = 0.85), while the combined IL-6 + TNF-*α* + β₂-MG model achieved superior discrimination (AUC = 0.91). Higher biomarker levels were associated with shorter relapse-free survival in Cox models, including high IL-6 (HR = 1.78), TNF-*α* (HR = 1.52), and β₂-MG (HR = 2.06). Dynamic changes also showed strong predictive value, with ΔIL-6 (OR = 1.21) and Δβ₂-MG (OR = 1.34) indicating early biological progression before radiologic relapse becomes detectable.

**Conclusion:**

Longitudinal monitoring of IL-6, TNF-*α*, and β₂-MG identifies early inflammatory trajectories linked to lymphoma relapse. IL-6 and β₂-MG at 3 months post-chemotherapy are independent and robust predictors, and a multi-marker model markedly enhances early-warning performance.

## Introduction

Lymphoma represents one of the most common hematologic malignancies, encompassing both Hodgkin lymphoma (HL) and non-Hodgkin lymphoma (NHL) (1, 2). Among NHL subtypes, diffuse large B-cell lymphoma (DLBCL) is the most frequently diagnosed individual entity (3). Although immunochemotherapy regimens such as R-CHOP have substantially improved overall survival, 30–40% of patients still experience relapse or develop refractory disease after frontline treatment (4–6). Relapse remains a major clinical challenge. Therapeutic options after recurrence are limited, often costly, and associated with considerable toxicity (7, 8). Moreover, survival outcomes decline sharply following relapse, particularly among patients who recur within the first year after chemotherapy (9, 10). Therefore, accurately identifying patients at high risk of relapse after completing initial treatment is critical for optimizing follow-up strategies, guiding early intervention, and improving long-term prognosis (11).

Current post-treatment surveillance of lymphoma relies predominantly on imaging modalities such as CT and PET-CT (9, 10, 12). Despite their status as the clinical standard, imaging modalities are limited by long intervals, high cost and radiation, and inadequate sensitivity for detecting minimal residual disease (MRD) (13–15). As a result, early biochemical signs of recurrence may remain undetected until radiologic progression becomes evident (7, 8). Routine hematologic indicators, such as lactate dehydrogenase (LDH) and peripheral lymphocyte counts, provide limited predictive value because they are nonspecific, influenced by diverse physiological and inflammatory conditions, and typically rise only after clinically or imaging-confirmed relapse (16–18). Thus, there is a clear need for reliable and low-cost biomarkers that can dynamically signal tumor activity and enable earlier relapse detection (19, 20).

Inflammation plays a central role in lymphoma biology, and several circulating biomarkers may capture early tumor-driven immune dysregulation (21, 22). IL-6 is a key pro-inflammatory cytokine, promoting tumor cell proliferation, survival, and immune evasion; elevated IL-6 levels have been consistently associated with poor prognosis in lymphoma (23, 24). TNF-*α*, another critical inflammatory mediator, reflects activation of the tumor microenvironment and B-cell responses, and may serve as an indicator of immune imbalance and tumor burden (25–27). β₂-microglobulin (β₂-MG), a structural component of MHC class I, is one of the most established prognostic markers in lymphoid malignancies and reflects cellular turnover, tumor load, and immune activation (28, 29). Given their biological relevance and consistent associations with lymphoma progression, these three biomarkers represent promising candidates for sensitive and dynamic monitoring of relapse risk.

Although IL-6, TNF-*α*, and β₂-MG have been individually linked to lymphoma prognosis, several important gaps remain (24, 25, 27, 30, 31). Most existing studies are cross-sectional or assess only a single time point, making it impossible to evaluate dynamic biomarker changes in relation to relapse (26, 28, 29). Furthermore, the evidence is dominated by retrospective analyses, with little continuous monitoring during the critical post-chemotherapy period when relapse begins to emerge (23, 27). Consequently, it remains unclear whether early post-treatment biomarker trajectories can predict relapse and whether combined biomarker panels outperform single indicators. This study prospectively evaluated longitudinal changes in IL-6, TNF-*α*, and β₂-MG at key post-treatment time points to determine their association with lymphoma relapse. By assessing both absolute levels and dynamic trajectories, we aimed to identify whether early post-chemotherapy fluctuations provide actionable early warning signals.

## Methods

### Study design

This prospective cohort study was conducted at The Second Hospital of Hebei Medical University between January 2019 and December 2023. A total of 260 patients with pathologically confirmed lymphoma who had completed standard first-line chemotherapy were enrolled and followed longitudinally. Based on clinical outcomes during follow-up, patients were classified into a relapse group (*n* = 78) and a non-relapse group (*n* = 182).

The study protocol was reviewed and approved by the Ethics Committee of The Second Hospital of Hebei Medical University. Written informed consent was obtained from all participants prior to enrollment. All study procedures adhered to the principles of the Declaration of Helsinki.

### Sample size estimation

The sample size was determined based on the anticipated relapse rate and the requirements of multivariable logistic regression. Assuming an expected 2-year relapse rate of approximately 30%, at least 80 relapse events would be needed to satisfy the rule of ≥10 events per variable for stable model estimation. Accordingly, the target sample size was set at 250–300 patients, which was deemed sufficient to ensure adequate statistical power for both logistic and survival analyses.

### Inclusion criteria and exclusion criteria

Patients were eligible for enrollment if they met all of the following criteria: (1) Age ≥18 years; (2) Pathologically confirmed non-Hodgkin lymphoma (NHL), including diffuse large B-cell lymphoma (DLBCL), T-cell lymphoma, or other NHL subtypes; (3) Completion of a standard first-line chemotherapy regimen (e.g., R-CHOP) with documented complete response (CR) or partial response (PR); (4) Ability and willingness to complete at least 24 months of clinical follow-up; (5) Availability of complete serum biomarker data at all predefined time points (T1–T3).

Patients were excluded if they met any of the following conditions: (1) Evidence of active infection, autoimmune disease, or chronic inflammatory conditions during chemotherapy or within 3 months before biomarker assessment; (2) Presence of other concurrent malignancies; (3) Severe hepatic or renal dysfunction, or a recent acute cardiovascular or cerebrovascular event; (4) Inability to comply with follow-up procedures or loss to follow-up.

### Data collection and follow-up

#### Follow-up time points

Patients were assessed at predefined time points before and after completion of chemotherapy to capture dynamic clinical and biochemical changes. Baseline measurements were obtained before chemotherapy initiation (T0) when available. However, cytokine measurements at T0 were not included in the primary analyses because baseline levels at diagnosis are subject to substantial variability related to tumor burden and systemic inflammatory activity, which may limit their specificity for relapse prediction.

The first post-treatment assessment (T1) was performed within 1 week after completion of chemotherapy. Subsequent follow-up evaluations were conducted at 3 months (T2) and 6 months (T3) after treatment. An additional time point, Tr, was defined for immediate assessment at the onset of clinically suspected relapse. These intervals were chosen to monitor early post-treatment biological fluctuations under relatively stable clinical conditions and to facilitate timely detection of relapse.

#### Follow-up assessments

At each scheduled follow-up visit, comprehensive clinical and laboratory evaluations were performed. These included a detailed physical examination and review of symptoms, as well as imaging studies such as contrast-enhanced CT, PET-CT, or MRI, in accordance with routine clinical practice. Standard hematologic parameters, including lactate dehydrogenase (LDH) and peripheral lymphocyte counts, were obtained at each visit. Serum concentrations of IL-6, TNF-*α*, and β₂-MG were measured at all designated time points (T1–T3). All relapse events were systematically documented, including the date of recurrence and the clinical or imaging criteria supporting the diagnosis.

#### Definition of relapse

Relapse was defined based on the Lugano 2014 classification, which incorporates both radiologic and clinical criteria. Recurrence was diagnosed when imaging revealed new lesions, progression of previously involved sites, or when biological evidence of lymphoma activity was confirmed through laboratory or pathological assessment. Relapse-free survival was calculated from the end of chemotherapy to the date of first confirmed relapse or the last follow-up visit.

### Biomarker measurement

#### Sample collection

Fasting venous blood samples were collected in the early morning at each predefined time point. After centrifugation, serum was immediately separated, aliquoted, and stored at −80 °C until analysis. To ensure sample integrity, repeated freeze–thaw cycles were strictly avoided, and all specimens were processed according to standardized laboratory procedures.

### Assay methods

Serum IL-6 and TNF-*α* concentrations were quantified using commercially available enzyme-linked immunosorbent assay (ELISA) kits. All ELISA plates were read using a SpectraMax iD5 microplate reader. Assay sensitivity was 0.70 pg./mL for IL-6 and 1.2 pg./mL for TNF-*α*, with manufacturer-reported detection ranges of 1.0–300 pg./mL and 2.0–500 pg./mL, respectively. For additional confirmation, parallel measurements were performed using chemiluminescence immunoassay kits based on the New Industry Chemiluminescence Analyzer MAGLWNI X8 platform. All calibrators and controls were run according to the standard Roche protocol. Serum β₂-MG levels were determined using a latex-enhanced immunoturbidimetric assay on a Roche Cobas 8000 automated biochemical analyzer. The analytical sensitivity was 0.1 mg/L, with a linear detection range of 0.1–10.0 mg/L. All assays were performed strictly following manufacturers’ instructions. Calibration was performed before each batch, and internal quality controls were included at low, medium, and high concentration levels.

### Quality control

All biomarker analyses were performed in the same certified clinical laboratory to minimize inter-laboratory variability. Both intra-assay and inter-assay coefficients of variation (CVs) were maintained below 10%. Each sample was measured in duplicate, and the mean value was used for analysis. Laboratory personnel conducting the assays were blinded to all clinical information, including relapse status and time points, to prevent measurement bias.

### Variable definitions

#### Independent variables

The primary independent variables were serum levels of IL-6, TNF-*α*, and β₂-MG measured at three predefined post-treatment time points: T1 (within 1 week after chemotherapy), T2 (3 months post-treatment), and T3 (6 months post-treatment). In addition to absolute concentrations, dynamic changes (*Δ* values) were calculated to capture temporal biomarker trajectories, defined as ΔT2–T1 = T2 − T1 and ΔT3–T2 = T3 − T2. These variables were used to evaluate whether early biomarker fluctuations were associated with subsequent relapse risk.

#### Covariates

Covariates included key demographic, clinical, laboratory, and treatment-related variables. Baseline characteristics comprised age, sex, and body mass index (BMI). Clinical covariates included lymphoma subtype, Ann Arbor stage, and International Prognostic Index (IPI) score. Laboratory parameters included lactate dehydrogenase (LDH) and peripheral lymphocyte count at baseline. Treatment-related factors included the chemotherapy regimen, the number of chemotherapy cycles completed, and whether the patient underwent autologous stem cell transplantation. These covariates were incorporated into multivariable analyses to control for potential confounding.

#### Outcome variables

The primary outcome was disease relapse, defined as a binary variable (Yes/No) according to the Lugano 2014 criteria. The secondary outcome was time to relapse, measured from the end of chemotherapy to the date of confirmed recurrence or last follow-up. This time-to-event variable was used for survival analyses, including Kaplan–Meier estimation and Cox proportional hazards modeling.

### Statistical analysis

Continuous variables were assessed for normality and summarized as mean ± standard deviation for normally distributed data or as median (interquartile range) when distributional assumptions were not met. Categorical variables were presented as counts and percentages. Missing data were minimal; when the proportion of missing values was less than 5%, mean or median imputation was applied as appropriate. Variables with more than 5% missingness were documented and handled as described in the Appendix.

Baseline differences between the relapse and non-relapse groups were evaluated using the independent-sample *t* test or Mann–Whitney *U* test for continuous variables, depending on data distribution, and the χ^2^ test or Fisher’s exact test for categorical variables.

Univariate logistic regression analyses were performed to evaluate the associations between each biomarker (IL-6, TNF-*α*, and β₂-microglobulin) measured at T1, T2, and T3 and the risk of relapse. Dynamic changes in biomarker levels (ΔT2–T1 and ΔT3–T2) were also examined. Multivariable logistic regression models were subsequently constructed focusing on biomarker levels at T2, given their strongest temporal association with relapse. IL-6, TNF-*α*, and β₂-microglobulin at T2 were entered simultaneously along with key clinical covariates. Results were reported as odds ratios (ORs) with 95% confidence intervals (CIs). Multicollinearity was assessed using variance inflation factors (VIFs); because dynamic change variables showed substantial collinearity with absolute T2 values, they were included only in univariate analyses.

Receiver operating characteristic (ROC) curves were generated to evaluate the discriminative performance of individual biomarkers at T2. A combined model incorporating IL-6, TNF-*α*, and β₂-microglobulin was also developed. The area under the ROC curve (AUC), sensitivity, specificity, and optimal cutoff values determined by the Youden index were calculated. Comparisons between AUCs were performed using DeLong’s test.

Relapse-free survival (RFS) was analyzed using the Kaplan–Meier method after stratifying patients into high- and low-biomarker groups based on median T2 values. Differences in survival distributions were assessed using the log-rank test. Multivariable Cox proportional hazards regression models were then fitted to examine the associations between T2 biomarker levels and RFS, adjusting for clinically relevant covariates including Ann Arbor stage, International Prognostic Index (IPI) score, and autologous stem cell transplantation status. Hazard ratios (HRs) with 95% CIs were reported, and the proportional hazards assumption was evaluated using Schoenfeld residuals.

To assess potential overfitting and model stability, internal validation was performed using repeated five-fold cross-validation. Model discrimination was summarized using the mean cross-validated AUC. Given the single-center nature of the cohort, orthogonal validation using an independent external dataset was not feasible; therefore, model performance is reported as internally validated discrimination rather than externally validated prediction.

All statistical analyses were conducted using R software (version 4.x; R Foundation for Statistical Computing) and SPSS (version 26.0; IBM Corp., Armonk, NY). ROC, Kaplan–Meier, and Cox regression analyses were implemented using the *pROC*, *survival*, and *rms* packages in R. All tests were two-sided, and a *p* value < 0.05 was considered statistically significant.

## Results

### Baseline characteristics

A total of 260 patients were included in the final analysis, of whom 78 (30.0%) experienced relapse during follow-up. The baseline characteristics of the study population are summarized in Table 1. The cohort comprised patients with clearly defined histopathological subtypes, including diffuse large B-cell lymphoma (DLBCL), T-cell lymphomas, follicular lymphoma, marginal zone lymphoma, and mantle cell lymphoma.

**Table 1:** Baseline characteristics of the study population.

Characteristic	Total (*n* = 260)	Relapse (*n* = 78)	Non-relapse (*n* = 182)	*p* value
Age, years, mean ± SD	55.72 ± 11.70	53.20 ± 12.10	56.80 ± 11.50	0.045
Male sex, *n* (%)	150 (57.69)	50 (64.10)	100 (54.95)	0.180
BMI, kg/m^2^, mean ± SD	24.01 ± 3.05	23.80 ± 3.20	24.10 ± 3.00	0.421
Pathological subtype, *n* (%)				**0.210**
Diffuse large B-cell lymphoma	150 (57.69)	50 (64.10)	100 (54.95)	
T-cell lymphomas^†^	50 (19.23)	15 (19.23)	35 (19.23)	
Follicular lymphoma	30 (11.54)	8 (10.26)	22 (12.09)	
Marginal zone lymphoma	18 (6.92)	5 (6.41)	13 (7.14)	
Mantle cell lymphoma	12 (4.62)	2 (2.56)	10 (5.49)	
Ann Arbor stage III–IV, *n* (%)	150 (57.69)	60 (76.92)	90 (49.45)	<**0.001**
High IPI score (3–5), *n* (%)	105 (40.38)	45 (57.69)	60 (32.97)	<**0.001**
B symptoms, *n* (%)	90 (34.62)	40 (51.28)	50 (27.47)	<**0.001**
LDH, U/L, mean ± SD	268.43 ± 88.50	310.50 ± 95.30	250.40 ± 80.20	**0.002**
β₂-microglobulin, mg/L, mean ± SD	3.34 ± 1.08	3.90 ± 1.20	3.10 ± 1.00	**0.003**
Lymphocyte count, ×10^9^/L, mean ± SD	1.43 ± 0.49	1.25 ± 0.45	1.50 ± 0.50	**0.010**
R-CHOP–based regimen, *n* (%)	215 (82.69)	60 (76.92)	155 (85.16)	0.120
Number of chemotherapy cycles, mean ± SD	6.34 ± 0.87	6.20 ± 1.00	6.40 ± 0.80	0.083
Autologous stem cell transplantation, *n* (%)	80 (30.77)	20 (25.64)	60 (32.97)	0.230

Baseline characteristics of the enrolled lymphoma patients. Continuous variables are presented as mean ± standard deviation, and categorical variables as number (percentage). *p* values were calculated using the independent-sample *t* test or chi-square test, as appropriate. ^†^T-cell lymphomas include peripheral T-cell lymphoma, not otherwise specified, and other mature T-cell neoplasms diagnosed according to contemporary WHO/ICC criteria. *P*<0.05, statistically significant difference.

Compared with the non-relapse group, patients who experienced relapse had a significantly higher proportion of advanced Ann Arbor stage III–IV disease (76.92% vs. 49.45%, *p* = 0.001), high International Prognostic Index (IPI) scores (57.69% vs. 32.97%, *p* = 0.001), and B symptoms (51.28% vs. 27.47%, *p* = 0.001). Biochemical indicators also differed significantly between groups. The relapse group exhibited higher baseline lactate dehydrogenase (LDH) levels (310.50 ± 95.30 vs. 250.40 ± 80.20 U/L, *p* = 0.002) and β₂-microglobulin levels (3.90 ± 1.20 vs. 3.10 ± 1.00 mg/L, *p* = 0.003), whereas lymphocyte counts were significantly lower (1.25 ± 0.45 vs. 1.50 ± 0.50 × 10^9^/L, *p* = 0.010).

The relapse group was slightly younger than the non-relapse group (53.20 ± 12.10 vs. 56.80 ± 11.50 years), with a marginal but statistically significant difference (*p* = 0.045). No significant differences were observed in sex distribution, body mass index, chemotherapy regimen, number of chemotherapy cycles, or autologous stem cell transplantation.

### Serum biomarker levels at different time points

Serum levels of IL-6, TNF-*α*, and β₂-MG at each assessment time point are presented in Table 2 and Supplementary Figure S1. At the end of chemotherapy (T1), all three biomarkers were significantly elevated in the relapse group compared with the non-relapse group, including IL-6 (12.73 ± 4.18 vs. 9.47 ± 3.31 pg./mL, *p* < 0.001), TNF-*α* (19.63 ± 6.33 vs. 15.27 ± 5.14 pg./mL, *p* < 0.001), and β₂-MG (3.87 ± 1.17 vs. 3.12 ± 0.97 mg/L, *p* = 0.002). These differences became more pronounced at the 3-month follow-up (T2), with relapse patients showing further increases in IL-6 (15.84 ± 4.96 vs. 10.36 ± 3.44 pg./mL), TNF-α (23.92 ± 7.18 vs. 16.51 ± 5.48 pg./mL), and β₂-MG (4.54 ± 1.28 vs. 3.27 ± 1.05 mg/L), all with *p* < 0.001. By the 6-month time point (T3), the divergence between groups was most marked. IL-6 continued to rise to 18.59 ± 5.47 pg./mL in the relapse group compared with 11.12 ± 3.87 pg./mL in the non-relapse group (*p* < 0.001). Similarly, TNF-*α* (27.84 ± 7.82 vs. 17.46 ± 6.12 pg./mL, *p* < 0.001) and β₂-MG (5.16 ± 1.41 vs. 3.34 ± 1.08 mg/L, *p* < 0.001) remained significantly higher in patients who later experienced relapse.

**Table 2:** Serum levels of IL-6, TNF-α, and β₂-microglobulin at different time points.

Biomarker	Time point	Relapse (*n* = 78)	Non-relapse (*n* = 182)	*p* value
IL-6 (pg/mL)	T1	12.73 ± 4.18	9.47 ± 3.31	<0.001
	T2	15.84 ± 4.96	10.36 ± 3.44	<0.001
	T3	18.59 ± 5.47	11.12 ± 3.87	<0.001
TNF-α (pg/mL)	T1	19.63 ± 6.33	15.27 ± 5.14	<0.001
	T2	23.92 ± 7.18	16.51 ± 5.48	<0.001
	T3	27.84 ± 7.82	17.46 ± 6.12	<0.001
β₂-microglobulin (mg/L)	T1	3.87 ± 1.17	3.12 ± 0.97	0.002
	T2	4.54 ± 1.28	3.27 ± 1.05	<0.001
	T3	5.16 ± 1.41	3.34 ± 1.08	<0.001

### Association between serum biomarkers and relapse

As shown in Table 3, IL-6, TNF-*α*, and β₂-MG measured at the 3-month follow-up (T2) were significantly associated with relapse in univariate analyses. Each 1-pg/mL increase in IL-6 at T2 was associated with a 12% higher odds of relapse (OR = 1.12, 95% CI: 1.08–1.17, *p* < 0.001), while TNF-α showed a smaller but significant effect (OR = 1.06, 95% CI: 1.03–1.09, *p* < 0.001). β₂-MG at T2 demonstrated the strongest association, with an OR of 1.55 (95% CI: 1.28–1.88, *p* < 0.001). Interval changes from T1 to T2 were also predictive; for example, ΔIL-6 (T2–T1) had an OR of 1.21 (95% CI: 1.11–1.32, *p* < 0.001).

**Table 3:** Univariate and multivariate logistic regression analyses for predicting relapse.

Variable	Univariate OR (95% CI)	*p* value	Multivariate OR (95% CI)	*p* value
IL-6 at T2 (per 1 pg/mL)	1.12 (1.08–1.17)	<0.001	1.08 (1.03–1.14)	0.001
TNF-α at T2 (per 1 pg/mL)	1.06 (1.03–1.09)	<0.001	1.03 (0.99–1.07)	0.118
β₂-microglobulin at T2 (per 1 mg/L)	1.55 (1.28–1.88)	<0.001	1.38 (1.11–1.72)	0.003
ΔIL-6 (T2–T1)	1.21 (1.11–1.32)	<0.001	—	—
Δβ₂-microglobulin (T2–T1)	1.34 (1.12–1.60)	0.001	—	—
Ann Arbor stage III–IV	2.40 (1.45–3.97)	0.001	1.71 (1.00–2.93)	0.049
High IPI score (3–5)	2.20 (1.35–3.58)	0.002	1.86 (1.09–3.19)	0.023
Autologous transplantation (Yes)	0.78 (0.45–1.35)	0.374	0.82 (0.45–1.49)	0.517
Age (per 1 year)	1.01 (0.99–1.03)	0.242	1.00 (0.98–1.03)	0.811
Sex (Male)	1.32 (0.80–2.16)	0.278	—	—

Univariate and multivariate logistic regression analyses for predictors of relapse. Odds ratios (ORs) are presented with 95% confidence intervals. Biomarkers measured at the 3-month follow-up (T2) were selected for multivariate analysis because they exhibited the strongest temporal association with relapse and demonstrated lower collinearity compared with T1 and T3 values. Interval change variables (ΔT2–T1) were included only in univariate analyses due to collinearity with absolute T2 measurements. In the final adjusted model, IL-6 (T2), β₂-microglobulin (T2), advanced Ann Arbor stage, and high IPI score remained independent predictors of relapse, whereas TNF-α (T2) lost significance after adjustment.

In the multivariate model (Table 3), IL-6 and β₂-MG at T2 remained independent predictors after adjustment for clinical covariates. IL-6 at T2 retained a significant effect with an adjusted OR of 1.08 (95% CI: 1.03–1.14, *p* = 0.001), and β₂-MG showed an adjusted OR of 1.38 (95% CI: 1.11–1.72, *p* = 0.003). In contrast, TNF-*α* at T2 was no longer significant (adjusted OR = 1.03, *p* = 0.118). Advanced Ann Arbor stage (III–IV) and high IPI score also remained independent risk factors with adjusted ORs of 1.71 (95% CI: 1.00–2.93, *p* = 0.049) and 2.14 (95% CI: 1.25–3.67, *p* = 0.023), respectively.

### Subtype-specific analyses in diffuse large B-cell lymphoma

Of the 260 patients included in the study, 150 (57.7%) were diagnosed with diffuse large B-cell lymphoma (DLBCL), representing the most prevalent histological subtype in the cohort. Within the DLBCL subgroup, serum levels of IL-6, TNF-*α*, andβ₂-MG measured at the 3-month post-treatment time point (T2) were significantly higher in patients who subsequently experienced relapse compared with those who remained relapse-free (Supplementary Table S1). After multivariable adjustment using the same covariate set as in the primary analysis—including age, Ann Arbor stage, International Prognostic Index (IPI) score, and autologous stem cell transplantation—IL-6 at T2 (adjusted OR = 1.07, 95% CI: 1.02–1.13) and β₂-MG at T2 (adjusted OR = 1.33, 95% CI: 1.05–1.69) remained independently associated with relapse risk, whereas TNF-*α* at T2 did not retain statistical significance (Supplementary Table S2).

### Diagnostic performance of biomarkers and the combined model

**Figure 1:**
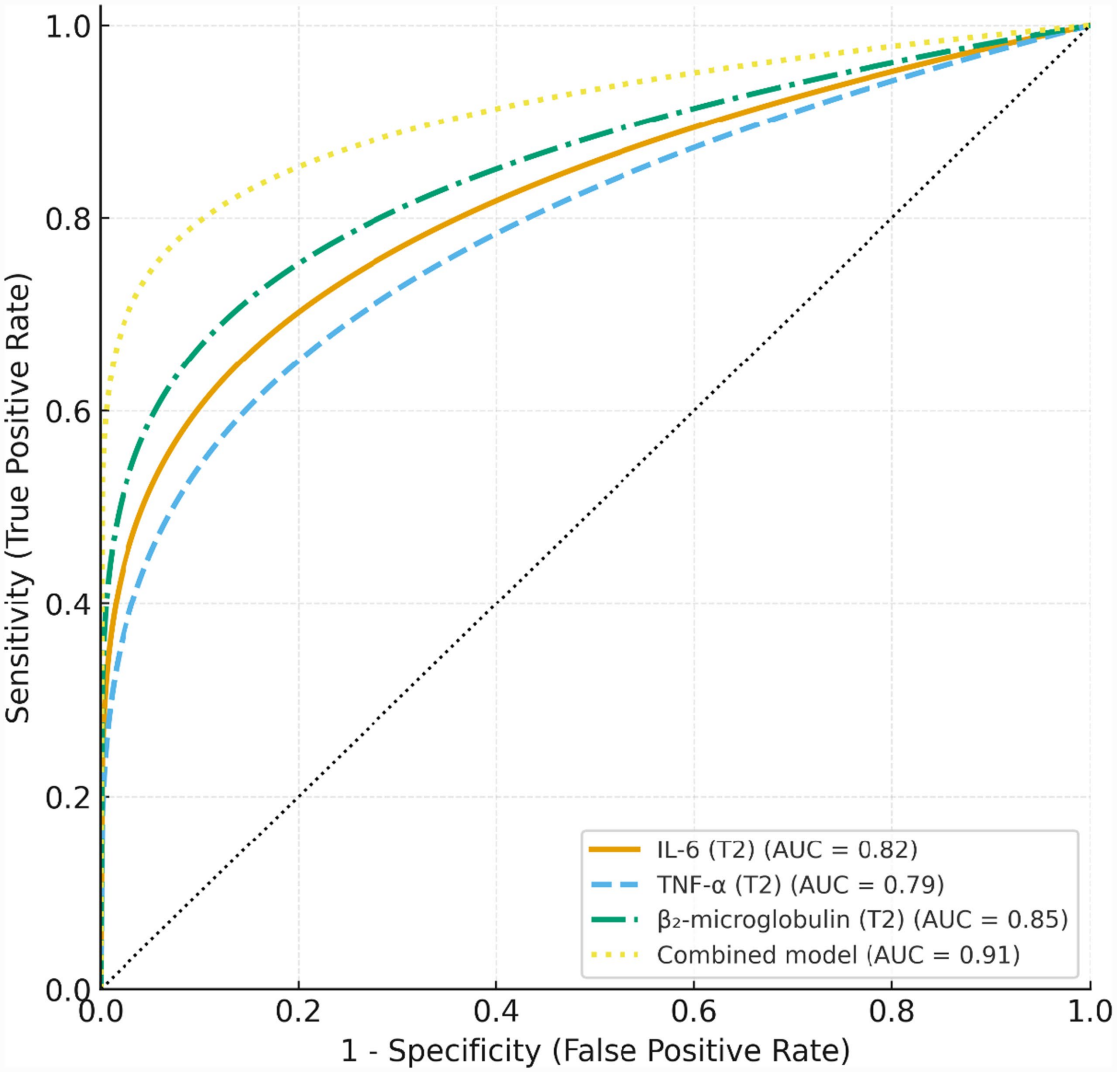
ROC curves for individual biomarkers and the combined model in predicting relapse. Receiver operating characteristic (ROC) curves for IL-6, TNF-α, β₂-microglobulin, and the combined biomarker model at the 3-month follow-up (T2).

As shown in Figure 1 and Table 4, all three biomarkers measured at the 3-month follow-up (T2)—IL-6, TNF-*α*, and β₂-MG—demonstrated significant diagnostic ability for predicting relapse. β₂-MG yielded the highest performance among the individual markers (AUC = 0.85, 95% CI: 0.79–0.90, *p* < 0.001), followed by IL-6 (AUC = 0.82, 95% CI: 0.76–0.88) and TNF-α (AUC = 0.79, 95% CI: 0.72–0.85). Sensitivity and specificity for single biomarkers ranged from 70.5 to 77.0% and 75.8 to 80.2%, respectively. The combined biomarker model demonstrated markedly superior discriminative power, achieving an AUC of 0.91 (95% CI: 0.87–0.95, *p* < 0.001), with improved sensitivity (84.6%) and specificity (86.3%).

**Table 4:** Diagnostic performance of individual biomarkers and the combined model.

Model	AUC (95% CI)	*p* value	Sensitivity (%)	Specificity (%)
IL-6 (T2)	0.82 (0.76–0.88)	<0.001	74.4	78.0
TNF-α (T2)	0.79 (0.72–0.85)	<0.001	70.5	75.8
β₂-microglobulin (T2)	0.85 (0.79–0.90)	<0.001	77.0	80.2
Combined model	0.91 (0.87–0.95)	<0.001	84.6	86.3

Diagnostic performance of individual biomarkers and the combined model for predicting relapse. AUC values are presented with 95% confidence intervals.

Similar discriminative patterns were observed when the analyses were restricted to patients with DLBCL, with the combined biomarker model consistently demonstrating superior predictive performance compared with individual biomarkers (Supplementary Table S3).

In repeated five-fold cross-validation, the combined biomarker model demonstrated stable discriminative performance, with a mean cross-validated AUC of 0.88, which was comparable to the apparent AUC estimated in the full cohort (Supplementary Table S4).

In addition to AUC, classification performance was evaluated using confusion matrices and recall-based metrics. At the optimal cutoff, the combined biomarker model demonstrated balanced sensitivity and specificity for relapse detection, indicating that discriminative performance was not driven solely by correct classification of the majority non-relapse group (Supplementary Table S5).

### Relapse-free survival analysis

Kaplan–Meier analyses demonstrated clear separation of relapse-free survival between high and low biomarker groups (Supplementary Figures S2A–C). Patients with high IL-6 levels at the 3-month assessment (T2) exhibited significantly shorter relapse-free survival compared with those with low IL-6 (log-rank *p* < 0.01). A similar pattern was observed for TNF-*α* and β₂-MG, with high-level groups showing a more rapid decline in survival probability over time. Among the three biomarkers, β₂-MG showed the widest curve separation, indicating the strongest prognostic impact.

In the multivariable Cox proportional hazards model (Table 5), high IL-6 (adjusted HR = 1.78, 95% CI: 1.20–2.64), high TNF-*α* (HR = 1.52, 95% CI: 1.03–2.26), and high β₂-MG (HR = 2.06, 95% CI: 1.36–3.11) remained independent predictors of earlier relapse after adjustment for Ann Arbor stage, IPI score, autologous transplantation, and age.

**Table 5:** Adjusted cox regression analysis for relapse-free survival.

Variable	Adjusted HR (95% CI)	*p* value
High IL-6 (vs. Low)	1.78 (1.20–2.64)	0.004
High TNF-α (vs. Low)	1.52 (1.03–2.26)	0.035
High β₂-microglobulin (vs. Low)	2.06 (1.36–3.11)	<0.001
Ann Arbor stage III–IV	1.63 (1.05–2.53)	0.029
High IPI score (3–5)	1.71 (1.11–2.63)	0.016
Autologous transplantation (Yes)	0.89 (0.52–1.53)	0.676
Age (per 1-year increase)	1.01 (0.99–1.04)	0.284

Multivariate Cox proportional hazards analysis of relapse-free survival. All models were adjusted for Ann Arbor stage (III–IV vs I–II), International Prognostic Index (IPI) score, autologous stem cell transplantation status, and age. Biomarkers were dichotomized into high and low groups based on median values at the 3-month follow-up (T2). HR >1 indicates increased risk of relapse.

In subgroup analyses limited to DLBCL, higher levels of IL-6 and β₂-MG at T2 were also associated with significantly shorter relapse-free survival, as demonstrated by Kaplan–Meier analyses (Supplementary Figure S3).

## Discussion

In this prospective cohort study, we had revealed several notable findings. First, all three biomarkers—IL-6, TNF-*α*, and β₂-MG—showed significant differences between relapse and non-relapse groups immediately after chemotherapy (T1). These differences widened at T2 and T3, driven by a progressive increase in the relapse group. The 3-month time point (T2) demonstrated the strongest predictive value: IL-6 and β₂-MG remained independent predictors in multivariable models, whereas TNF-α lost significance after adjustment. The combined biomarker model achieved superior discrimination (AUC = 0.91). Survival analyses further confirmed that higher IL-6, TNF-α, and β₂-MG were associated with shorter relapse-free survival. Overall, IL-6 and β₂-MG at 3 months post-chemotherapy independently predicted relapse within 12–24 months, and a multi-marker panel provided the strongest early-warning capacity.

Our findings align with prior evidence showing that IL-6 correlates with tumor burden and adverse outcomes in lymphoma (23, 26, 32, 33); however, most existing studies focus on diagnostic or static prognostic assessments (34, 35). In contrast, our results highlight the early post-treatment time point (T2) as a particularly informative window for relapse prediction, which has not been emphasized previously. For TNF-*α*, the literature has reported inconsistent predictive value, often weaker than IL-6 (25–27, 36). Our study supports this view: TNF-α was significant in univariate analyses but lost significance in multivariable models, likely due to collinearity with IL-6 (37–39). Consistent with its well-established prognostic role, β₂-MG again proved to be a strong predictor (30, 40–44). Notably, we demonstrate that β₂-MG shows superior discriminative ability for relapse compared with the other biomarkers (AUC = 0.85). Importantly, most previous studies have evaluated single biomarkers or cross-sectional data (31, 45, 46). Our study is the first to integrate longitudinal T1–T3 measurements with multiple analytic frameworks, demonstrating that a multi-marker model substantially enhances predictive performance.

Lymphoma comprises a biologically and clinically heterogeneous group of malignancies, and analyses that pool different histological subtypes may potentially obscure subtype-specific associations. To address this concern, we conducted a dedicated subgroup analysis focusing on patients with diffuse large B-cell lymphoma, the most common subtype in our cohort. Importantly, the key findings observed in the overall population—namely, the independent prognostic value of IL-6 and β₂-MG measured at 3 months after completion of chemotherapy—were preserved within the DLBCL subgroup. These results support the robustness and clinical relevance of our primary conclusions. Nevertheless, subgroup analyses of less frequent lymphoma subtypes were underpowered in the present study and should be interpreted with caution, warranting further validation in larger, subtype-specific cohorts.

Although internal validation demonstrated stable model performance, orthogonal validation in an independent cohort was not available in the present study. As a result, the findings should be interpreted as internally validated associations and discrimination. External validation in independent, preferably multicenter, cohorts will be required to confirm generalizability and to support potential clinical implementation.

The biological behavior of the three biomarkers provides a plausible explanation for their predictive value in relapse surveillance. IL-6 is a key pro-inflammatory cytokine that activates the JAK/STAT3 pathway, sustaining inflammatory signaling within the tumor microenvironment and promoting tumor cell proliferation, survival, and immune evasion (47, 48). Elevated IL-6 levels have been linked to increased proliferative activity and higher residual tumor burden, consistent with its strong association with relapse in our cohort (49, 50). TNF-*α* also contributes to lymphoma biology through activation of the NF-κB pathway, amplifying inflammatory cascades and reflecting dysregulated immune responses (25, 51). However, its close biological overlap with IL-6 likely explains the attenuation of its independent predictive effect in multivariable models, suggesting that TNF-α may function more as a marker of generalized inflammatory activation rather than a specific driver of relapse (26, 36). β₂-MG, a structural component of the MHC class I complex, reflects both tumor cell turnover and tumor burden, and has long been recognized as an adverse prognostic factor in aggressive lymphomas (27, 40). Its strong performance in predicting relapse in our study is consistent with its role as a direct indicator of residual disease activity (30). Finally, the prognostic relevance of dynamic changes further supports a biological model in which persistent or rising inflammation after chemotherapy reflects ongoing microscopic disease.

These findings have clear clinical relevance. Serial measurement of IL-6, TNF-*α*, and β₂-MG provides an accessible early-warning system that can detect relapse risk during the intervals between routine imaging, offering particular value for patients who cannot undergo frequent CT or PET scans. The 3-month post-treatment time point (T2) emerges as a critical surveillance window, during which biomarker elevations may justify earlier imaging, shortened follow-up intervals, or consideration of intensified monitoring. The combined biomarker model further enables practical risk stratification, distinguishing low-risk patients who may continue standard follow-up from high-risk individuals who may benefit from closer monitoring or adjunctive MRD assessments such as ctDNA. Given their low cost and ease of implementation, these serum biomarkers represent a feasible and scalable complement—rather than an alternative—to conventional imaging-based surveillance.

This study has several limitations. First, it was conducted at a single center with a moderate sample size, which may limit the generalizability of the findings and warrants validation in larger multi-center cohorts. Second, molecular MRD markers such as ctDNA or flow cytometric MRD were not included; integrating these assays could further refine relapse prediction. Third, we did not perform subtype-specific analyses, and differences between lymphoma subtypes—such as DLBCL and T-cell lymphomas—may influence biomarker performance. In addition, the potential impact of immunomodulatory treatments, including PD-1 inhibitors, was not fully controlled for and may affect inflammatory marker levels. Although internal validation using repeated cross-validation demonstrated stable model performance, external validation in independent cohorts will be required before clinical implementation. Finally, the follow-up duration was relatively limited, and longer-term outcomes such as late relapse and overall survival require further investigation. Future research should aim to validate these findings in large, multi-center prospective cohorts to strengthen external applicability. Incorporating molecular MRD assays, particularly ctDNA, may enable the development of a multi-modal surveillance strategy that integrates inflammatory biomarkers with tumor-specific genetic markers. Subtype-specific modeling and exploration of treatment–biomarker interactions could also enhance predictive precision. Ultimately, combining dynamic serum biomarkers with molecular and imaging data may yield a more comprehensive, personalized relapse-monitoring framework.

## Conclusion

In this prospective cohort study, longitudinal changes in IL-6, TNF-*α*, and β₂-MG were associated with subsequent lymphoma relapse. IL-6 and β₂-MG measured at 3 months after chemotherapy demonstrated independent prognostic value, and a combined biomarker model improved discriminative performance compared with individual markers. Notably, biomarker dynamics preceded radiologic relapse, suggesting potential utility for early risk stratification. These findings, which were consistent in the diffuse large B-cell lymphoma subgroup, support the use of readily available serum biomarkers as adjuncts to imaging in post-treatment surveillance.

## Data Availability

The original contributions presented in the study are included in the article/Supplementary material, further inquiries can be directed to the corresponding author.

## References

[1] Yao Z Deng L Xu-Monette ZY Manyam GC Jain P Tzankov A . Concordant bone marrow involvement of diffuse large B-cell lymphoma represents a distinct clinical and biological entity in the era of immunotherapy. Leukemia. (2018) 32:353–63. doi: 10.1038/leu.2017.222, 28745330 PMC5985660

[2] van Bladel DAG Stevens WBC Kroeze LI de Groen RAL de Groot FA van der Last-Kempkes J . A significant proportion of classic Hodgkin lymphoma recurrences represents clonally unrelated second primary lymphoma. Blood Adv. (2023) 7:5911–24. doi: 10.1182/bloodadvances.2023010412, 37552109 PMC10558751

[3] Karmali R Gordon LI. Sequencing T-cell therapy for relapsed or refractory large B-cell lymphoma. Lancet Haematol. (2025) 12:946–55. doi: 10.1016/S2352-3026(25)00342-4 41448214

[4] Reitter S Rohn A Schmidt HH Linkesch W. Upfront radioimmunotherapy represents an effective and safe therapeutic option in cutaneous B-cell non-Hodgkin lymphoma. Ann Hematol. (2012) 91:129–30. doi: 10.1007/s00277-011-1216-1, 21437587

[5] Peng W Wu J Feng J. Long noncoding RNA HULC predicts poor clinical outcome and represents pro-oncogenic activity in diffuse large B-cell lymphoma. Biomed Pharmacother. (2016) 79:188–93. doi: 10.1016/j.biopha.2016.02.032, 27044827

[6] Manni S Pesavento M Spinello Z Saggin L Arjomand A Fregnani A . Protein kinase CK2 represents a new target to boost Ibrutinib and Venetoclax induced cytotoxicity in mantle cell lymphoma. Front Cell Dev Biol. (2022) 10:935023. doi: 10.3389/fcell.2022.935023, 36035991 PMC9403710

[7] Humme D Haider A Mobs M Mitsui H Suárez-Fariñas M Ohmatsu H . Aurora kinase a is upregulated in cutaneous T-cell lymphoma and represents a potential therapeutic target. J Invest Dermatol. (2015) 135:2292–300. doi: 10.1038/jid.2015.139, 25848977

[8] Hall JS Usher S Byers RJ Higgins RC Memon D Radford JA . QuantiGene plex represents a promising diagnostic tool for cell-of-origin subtyping of diffuse large B-cell lymphoma. J Mol Diagn. (2015) 17:402–11. doi: 10.1016/j.jmoldx.2015.03.010 25982535

[9] Dai L Lin Z Qiao J Chen Y Flemington EK Qin Z. Ribonucleotide reductase represents a novel therapeutic target in primary effusion lymphoma. Oncogene. (2017) 36:5068–74. doi: 10.1038/onc.2017.122, 28459467 PMC5578886

[10] Culpin RE Sieniawski M Proctor SJ Menon G Mainou-Fowler T. MicroRNAs are suitable for assessment as biomarkers from formalin-fixed paraffin-embedded tissue, and miR-24 represents an appropriate reference microRNA for diffuse large B-cell lymphoma studies. J Clin Pathol. (2013) 66:249–52. doi: 10.1136/jclinpath-2012-201021, 23172553

[11] Belmonte B Cancila V Gulino A Navari M Arancio W Macor P . Constitutive PSGL-1 correlates with CD30 and TCR pathways and represents a potential target for immunotherapy in anaplastic large T-cell lymphoma. Cancers. (2021) 13:2958. doi: 10.3390/cancers13122958, 34204843 PMC8231564

[12] Cui C Cao J Li Y Jia B Ma N Li X . A proof of concept study of (18)F-FDG PET/CT patient-level radiomics identify refractory/relapsed diffuse large B-cell lymphoma. Sci Rep. (2025) 15:33914. doi: 10.1038/s41598-025-08223-8, 41028770 PMC12484957

[13] Editorial Office. Erratum: integration of PET/CT parameters and a clinical variable to predict the risk of progression of disease within 24 months (POD24) in follicular lymphoma. Quant Imaging Med Surg. (2025) 15:10403–8. doi: 10.21037/qims-2025-01 41081141 PMC12514617

[14] Gibbs AC Batchala PP Gottlieb CE Balle BO Obiorah IE Foster LH . Staging FDG PET/CT prediction of bone marrow involvement by diffuse large B-cell lymphoma leading to delayed recognition of multiple myeloma. J Hematop. (2025) 18:49. doi: 10.1007/s12308-025-00667-1, 41186833 PMC12586389

[15] Habouzit V Peoc'h M Nathalie P Chalayer E Cathebras P. Concomitant diagnosis and therapeutic response of large-vessel Vasculitis and Hodgkin lymphoma on 18 F-FDG PET/CT. Clin Nucl Med. (2025) 50:1233–5. doi: 10.1097/RLU.0000000000006124, 40938194

[16] Le Du K Chauchet A Sadot-Lebouvier S Fitoussi O Fontanet B Saint-Lezer A . Comparison of electronic surveillance with routine monitoring for patients with lymphoma at high risk of relapse: prospective randomized controlled phase 3 trial (sentinel lymphoma). JMIR Cancer. (2025) 11:e65960. doi: 10.2196/65960, 40327037 PMC12070818

[17] Kimby E. Rituximab versus active surveillance in patients with follicular lymphoma. Lancet Haematol. (2025) 12:e320–1. doi: 10.1016/S2352-3026(25)00105-X, 40306823

[18] Kawasaki A Matsumoto I Izutsu K Nishikawa R. Post-marketing surveillance of tirabrutinib in 189 patients with r/r primary central nervous system lymphoma. Future Oncol. (2025) 21:1837–47. doi: 10.1080/14796694.2025.2507561, 40421905 PMC12150651

[19] Kawasaki A Hatake K Matsumura I Izutsu K Hoshino T Akamatsu A . Post-marketing surveillance of the safety and effectiveness of nivolumab for classic Hodgkin lymphoma in Japan. Int J Hematol. (2024) 119:667–76. doi: 10.1007/s12185-024-03734-y, 38521840 PMC11136857

[20] Ishitsuka K Yasukawa T Tsuji Y. Safety and effectiveness of mogamulizumab in relapsed or refractory CC chemokine receptor 4-positive peripheral T-cell lymphoma and relapsed or refractory cutaneous T-cell lymphoma: a post-marketing surveillance in Japan. Hematol Oncol. (2024) 42:e3292. doi: 10.1002/hon.3292, 38847317

[21] Zhang H Xu L Yang L Su Z Kang H Xie X . Deep learning-based intratumoral and peritumoral features for differentiating ocular adnexal lymphoma and idiopathic orbital inflammation. Eur Radiol. (2025) 35:1276–89. doi: 10.1007/s00330-024-11275-5 39702637

[22] Wang G Qu X Guo J Luo Y Xian J. Radiomics and machine learning model can improve the differentiation between ocular adnexal lymphoma and idiopathic orbital inflammation. Chin Med J. (2025) 138:234–6. doi: 10.1097/CM9.0000000000003356, 39474730 PMC11745851

[23] Zhu Q Li H Zheng S Wang B Li M Zeng W . IL-6 and IL-10 are associated with gram-negative and gram-positive bacteria infection in lymphoma. Front Immunol. (2022) 13:856039. doi: 10.3389/fimmu.2022.856039, 35432366 PMC9011156

[24] Wang Y Zheng N Sun T Zhao H Chen Y Liu C. Role of TGM2 in T-cell lymphoblastic lymphoma via regulation of IL-6/JAK/STAT3 signalling. Mol Med Rep. (2022) 25:12592. doi: 10.3892/mmr.2022.12592, 35014680 PMC8778669

[25] Nedoszytko B Olszewska B Roszkiewicz J Glen J Zabłotna M Ługowska-Umer H . The role of polymorphism of interleukin-2, −10, −13 and TNF-alpha genes in cutaneous T-cell lymphoma pathogenesis. Postepy Dermatol Alergol. (2016) 33:429–34. doi: 10.5114/ada.2016.63881 28035219 PMC5183781

[26] Mozas P Rivas-Delgado A Rivero A Dlouhy I Nadeu F Balagué O . High serum levels of IL-2R, IL-6, and TNF-α are associated with higher tumor burden and poorer outcome of follicular lymphoma patients in the rituximab era. Leuk Res. (2020) 94:106371. doi: 10.1016/j.leukres.2020.106371, 32473488

[27] Gupta U Hira SK Singh R Paladhi A Srivastava P Pratim Manna P. Essential role of TNF-α in gamma c cytokine aided crosstalk between dendritic cells and natural killer cells in experimental murine lymphoma. Int Immunopharmacol. (2020) 78:106031. doi: 10.1016/j.intimp.2019.106031, 31821938

[28] Kim HD Cho H Jeong H Kim H‐D Bang K Kim S . A prognostic index for extranodal marginal‐zone lymphoma based on the mucosa‐associated lymphoid tissue international prognostic index and serum β2‐microglobulin levels. Br J Haematol. (2021) 193:307–15. doi: 10.1111/bjh.17222, 33216979

[29] Kang J Yoon S Suh C. Relevance of prognostic index with beta2-microglobulin for patients with diffuse large B-cell lymphoma in the rituximab era. Blood Res. (2017) 52:276–84. doi: 10.5045/br.2017.52.4.276, 29333404 PMC5762738

[30] Zhao P Zhu L Song Z Wang X Ma W Zhu X . Combination of baseline total metabolic tumor volume measured on FDG‐PET/CT and β2‐microglobulin have a robust predictive value in patients with primary breast lymphoma. Hematol Oncol. (2020) 38:493–500. doi: 10.1002/hon.2763, 32533716

[31] Maeyama M Sasayama T Tanaka K Nakamizo S Tanaka H Nishihara M . Multi‐marker algorithms based on CXCL13, IL‐10, sIL‐2 receptor, and β2‐microglobulin in cerebrospinal fluid to diagnose CNS lymphoma. Cancer Med. (2020) 9:4114–25. doi: 10.1002/cam4.3048, 32314548 PMC7300423

[32] Wang P Wu Y Yang C Zhao G Liu Y Cheng G . Embelin promotes oncolytic vaccinia virus-mediated antitumor immunity through disruption of IL-6/STAT3 signaling in lymphoma. Onco Targets Ther. (2020) 13:1421–9. doi: 10.2147/OTT.S209312, 32110041 PMC7034962

[33] Rong Q Gao Y Cai Q Wang X Bai B Ping L . High IL-6 expression in the tumor microenvironment is associated with poor prognosis of patients with extranodal natural / killer T-cell lymphoma (ENKTL). Expert Rev Anticancer Ther. (2021) 21:121–7. doi: 10.1080/14737140.2021.1853531, 33397158

[34] Liu J Hong J Ahn KS Go J Han H Park J . ERK-dependent IL-6 positive feedback loop mediates resistance against a combined treatment using danusertib and BKM120 in Burkitt lymphoma cell lines. Leuk Lymphoma. (2019) 60:2532–40. doi: 10.1080/10428194.2019.1594211, 30947576

[35] Hashwah H Bertram K Stirm K Stelling A Wu CT Kasser S . The IL-6 signaling complex is a critical driver, negative prognostic factor, and therapeutic target in diffuse large B-cell lymphoma. EMBO Mol Med. (2019) 11:e10576. doi: 10.15252/emmm.201910576, 31515941 PMC6783642

[36] Hira SK Mondal I Bhattacharya D Gupta KK Manna PP. Downregulation of STAT3 phosphorylation enhances tumoricidal effect of IL-15-activated dendritic cell against doxorubicin-resistant lymphoma and leukemia via TNF-α. Int J Biochem Cell Biol. (2015) 67:1–13. doi: 10.1016/j.biocel.2015.08.002, 26255115

[37] Federico M Guglielmi C Luminari S Mammi C Marcheselli L Gianelli U . Prognostic relevance of serum 2 microglobulin in patients with follicular lymphoma treated with anthracycline-containing regimens. A GISL study. Haematologica. (2007) 92:1482–8. doi: 10.3324/haematol.11502, 18024396

[38] Fan Y Huang Y Zheng W Su Z Hu J Yuan S. Cerebrospinal fluid β2-microglobulin as a diagnostic biomarker in central nervous system lymphoma: a single-center retrospective analysis. Ann Hematol. (2025) 104:5711–8. doi: 10.1007/s00277-025-06719-x, 41212200 PMC12672816

[39] Duletic-Nacinovic A Stifter S Marijic B Lucin K Valković T Petranović D . Serum IL-6, IL-8, IL-10 and beta2-microglobulin in association with international prognostic index in diffuse large B cell lymphoma. Tumori. (2008) 94:511–7. doi: 10.1177/030089160809400412 18822687

[40] Zhong J Weng X Chen L. Prognostic value of serum beta2-microglobulin in predicting survival of patients with diffuse large B-cell lymphoma. Clin Lab. (2025) 71:240907. doi: 10.7754/Clin.Lab.2024.240907 40066559

[41] Zhang Z Chen K Yan L Yang Z Zhu Z Chen C . Low expression of dendritic cell-specific intercellular adhesion molecule-grabbing nonintegrin-related protein in non-Hodgkin lymphoma and significant correlations with lactic acid dehydrogenase and β2-microglobulin. Biochem Cell Biol. (2013) 91:214–20. doi: 10.1139/bcb-2012-0110, 23859015

[42] Yoo C Yoon DH Yoon S Kim S Huh J Park C-J . Prognostic impact of beta(2)-microglobulin in patients with non-gastric mucosa-associated lymphoid tissue lymphoma. Leuk Lymphoma. (2015) 56:688–93. doi: 10.3109/10428194.2014.917640 24913511

[43] Wang XL Wang XL He S Zhai HL. Association of beta2-microglobulin with the prognosis of non-Hodgkin's lymphoma: a meta analysis. Int J Clin Exp Med. (2015) 8:3992–3999. doi: 10.11648/j.ijcem.20150803.697 26064301 PMC4443135

[44] Tarakanova VL Suarez F Tibbetts SA Jacoby MA Weck KE Hess JL . Murine gammaherpesvirus 68 infection is associated with lymphoproliferative disease and lymphoma in BALB beta2 microglobulin-deficient mice. J Virol. (2005) 79:14668–79. doi: 10.1128/JVI.79.23.14668-14679.2005, 16282467 PMC1287585

[45] Tang X Jiang T Guo B Liu J Wang C Zhang Y . Serum β2-microglobulin level is associated with the survival of HIV-associated diffuse large B-cell lymphoma: a retrospective study from China. Sci Rep. (2025) 15:15870. doi: 10.1038/s41598-025-99888-8, 40335742 PMC12058983

[46] Kim HD Cho H Sohn BS Park C-S Huh J Ryu JS . Prognostic significance of serum beta2-microglobulin levels in patients with peripheral T-cell lymphoma not otherwise specified. Leuk Lymphoma. (2022) 63:124–30. doi: 10.1080/10428194.2021.1971220 34702115

[47] Gholiha AR Hollander P Glimelius I Hedstrom G Molin D Hjalgrim H . Revisiting IL-6 expression in the tumor microenvironment of classical Hodgkin lymphoma. Blood Adv. (2021) 5:1671–81. doi: 10.1182/bloodadvances.2020003664, 33720338 PMC7993098

[48] Evans BL Fenger JM Ballash G Brown M. Serum IL-6 and MCP-1 concentrations in dogs with lymphoma before and after doxorubicin treatment as a potential marker of cellular senescence. Vet Med Sci. (2022) 8:85–96. doi: 10.1002/vms3.633, 34655167 PMC8788977

[49] Di Napoli A Greco D Scafetta G Ascenzi F Gulino A Aurisicchio L . IL-10, IL-13, Eotaxin and IL-10/IL-6 ratio distinguish breast implant-associated anaplastic large-cell lymphoma from all types of benign late seromas. Cancer Immunol Immunother. (2021) 70:1379–92. doi: 10.1007/s00262-020-02778-3, 33146828 PMC8053183

[50] Bhethanabhotla S Tiwari A Sharma MC Vishnubhatla S Bakhshi S. Prognostic significance of IL-6 in Hodgkin lymphoma. Indian J Pediatr. (2019) 86:551–4. doi: 10.1007/s12098-019-02902-x, 30830568

[51] Al-Khatib SM Abdo N Al-Eitan LN Al-Mistarehi AH Zahran DJ Kewan TZ. The impact of IL-6 and IL-10 gene polymorphisms in diffuse large B-cell lymphoma risk and overall survival in an Arab population: a case-control study. Cancers (Basel). (2020) 12:382. doi: 10.3390/cancers12020382, 32046104 PMC7072608

