# Supplementary material for: Dynamic changes in serum IL-6, TNF-α, and β₂-microglobulin as early predictors of post-treatment relapse in lymphoma: a prospective cohort study
Source: Front Med (Lausanne). 2026 Feb 12;13:1750664. doi: 10.3389/fmed.2026.1750664 (PMC12935660; doi:10.3389/fmed.2026.1750664)
Supplement: Supplementary file 1 [file Table_1.DOCX]

**Supplementary Table S1**

**Baseline Characteristics of Patients With Diffuse Large B-Cell Lymphoma (DLBCL)**

| **Characteristic** | **Total (n = 150)** | **Relapse (n = 48)** | **Non-relapse (n = 102)** | **P value** |
| --- | --- | --- | --- | --- |
| Age, years | 55.6 ± 11.5 | 54.1 ± 11.8 | 56.3 ± 11.2 | 0.26 |
| Male sex, n (%) | 88 (58.7) | 32 (66.7) | 56 (54.9) | 0.18 |
| BMI, kg/m² | 24.0 ± 3.1 | 23.7 ± 3.3 | 24.2 ± 3.0 | 0.38 |
| Ann Arbor stage III–IV, n (%) | 92 (61.3) | 38 (79.2) | 54 (52.9) | **0.003** |
| High IPI score (3–5), n (%) | 62 (41.3) | 28 (58.3) | 34 (33.3) | **0.005** |
| B symptoms, n (%) | 56 (37.3) | 26 (54.2) | 30 (29.4) | **0.004** |
| LDH, U/L | 276.8 ± 86.4 | 318.6 ± 92.1 | 255.8 ± 78.4 | **0.002** |
| Baseline β₂-microglobulin, mg/L | 3.42 ± 1.07 | 3.95 ± 1.18 | 3.18 ± 0.96 | **0.003** |
| Lymphocyte count, ×10⁹/L | 1.41 ± 0.48 | 1.22 ± 0.44 | 1.48 ± 0.49 | **0.012** |
| R-CHOP–based regimen, n (%) | 122 (81.3) | 36 (75.0) | 86 (84.3) | 0.17 |
| Autologous stem cell transplantation, n (%) | 46 (30.7) | 12 (25.0) | 34 (33.3) | 0.29 |

**Table note:** Continuous variables are presented as mean ± standard deviation, and categorical variables as number (percentage). P values were calculated using the independent-sample *t* test or chi-square test, as appropriate.

**Supplementary Table S2**

**Univariate and Multivariable Logistic Regression Analyses for Relapse in Patients With DLBCL (T2 Biomarkers)**

| **Variable** | **Univariate OR (95% CI)** | **P value** | **Adjusted OR (95% CI)*** | **P value** |
| --- | --- | --- | --- | --- |
| IL-6 at T2 (per 1 pg/mL) | 1.11 (1.06–1.17) | <0.001 | **1.07 (1.02–1.13)** | **0.006** |
| TNF-α at T2 (per 1 pg/mL) | 1.05 (1.01–1.09) | 0.011 | 1.02 (0.98–1.07) | 0.31 |
| β₂-microglobulin at T2 (per 1 mg/L) | 1.49 (1.20–1.86) | <0.001 | **1.33 (1.05–1.69)** | **0.018** |
| Ann Arbor stage III–IV | 2.65 (1.35–5.18) | 0.004 | 1.68 (0.92–3.05) | 0.09 |
| High IPI score (3–5) | 2.63 (1.39–4.98) | 0.003 | **1.91 (1.04–3.50)** | **0.036** |
| Autologous stem cell transplantation (Yes) | 0.86 (0.41–1.81) | 0.69 | / | / |
| Age (per 1 year) | 1.01 (0.98–1.04) | 0.47 | / | / |

**Table note:** Odds ratios (ORs) are presented with 95% confidence intervals (CIs).
*Multivariable logistic regression models were adjusted for age, Ann Arbor stage, International Prognostic Index (IPI) score, and autologous stem cell transplantation status. Biomarkers were measured at the 3-month post-treatment time point (T2). Statistically significant results (*P* < 0.05) are shown in **bold**.

**Supplementary Table S3**

**Diagnostic Performance of Serum Biomarkers for Predicting Relapse in DLBCL (T2)**

| **Model** | **AUC (95% CI)** | **Sensitivity (%)** | **Specificity (%)** |
| --- | --- | --- | --- |
| IL-6 | 0.80 (0.72–0.87) | 72.9 | 76.5 |
| TNF-α | 0.77 (0.69–0.85) | 69.8 | 73.5 |
| β₂-microglobulin | 0.83 (0.76–0.90) | 75.0 | 79.4 |
| Combined model (IL-6 + TNF-α + β₂-MG) | **0.89 (0.83–0.94)** | **82.1** | **84.3** |

**Table note:** Receiver operating characteristic (ROC) curves were generated using biomarker levels measured at the 3-month post-treatment time point (T2). The combined model included IL-6, TNF-α, and β₂-microglobulin.

**Supplementary Table S4. Internal validation of biomarker models using repeated cross-validation**

| **Model** | **Apparent AUC (Full Cohort)** | **Cross-validated AUC (Mean)** | **ΔAUC** |
| --- | --- | --- | --- |
| **IL-6 at T2** | 0.82 | 0.80 | −0.02 |
| **TNF-α at T2** | 0.79 | 0.77 | −0.02 |
| **β₂-microglobulin at T2** | 0.85 | 0.83 | −0.02 |
| **Combined biomarker model** | 0.91 | 0.88 | −0.03 |

**Table note:***Apparent AUCs were estimated using the full cohort. Internal validation was performed using repeated five-fold cross-validation (100 repetitions). Cross-validated AUCs are reported as mean values across repetitions. ΔAUC indicates the difference between apparent and cross-validated performance.*

**Supplementary Table S5. Classification performance of the combined biomarker model at the optimal cutoff**

| **Category** | **Item** | **Value** |
| --- | --- | --- |
| **Confusion matrix** | Observed relapse → Predicted relapse | 66 |
|  | Observed relapse → Predicted non-relapse | 12 |
|  | Observed non-relapse → Predicted relapse | 25 |
|  | Observed non-relapse → Predicted non-relapse | 157 |
| **Performance metrics** | Sensitivity (Recall), % | 84.6 |
|  | Specificity, % | 86.3 |
|  | Positive predictive value (PPV), % | 72.5 |
|  | Negative predictive value (NPV), % | 92.9 |
|  | Overall accuracy, % | 85.8 |

**Table note: The optimal cutoff value was determined using the Youden index. Sensitivity (recall) represents the proportion of relapse cases correctly identified by the model.**


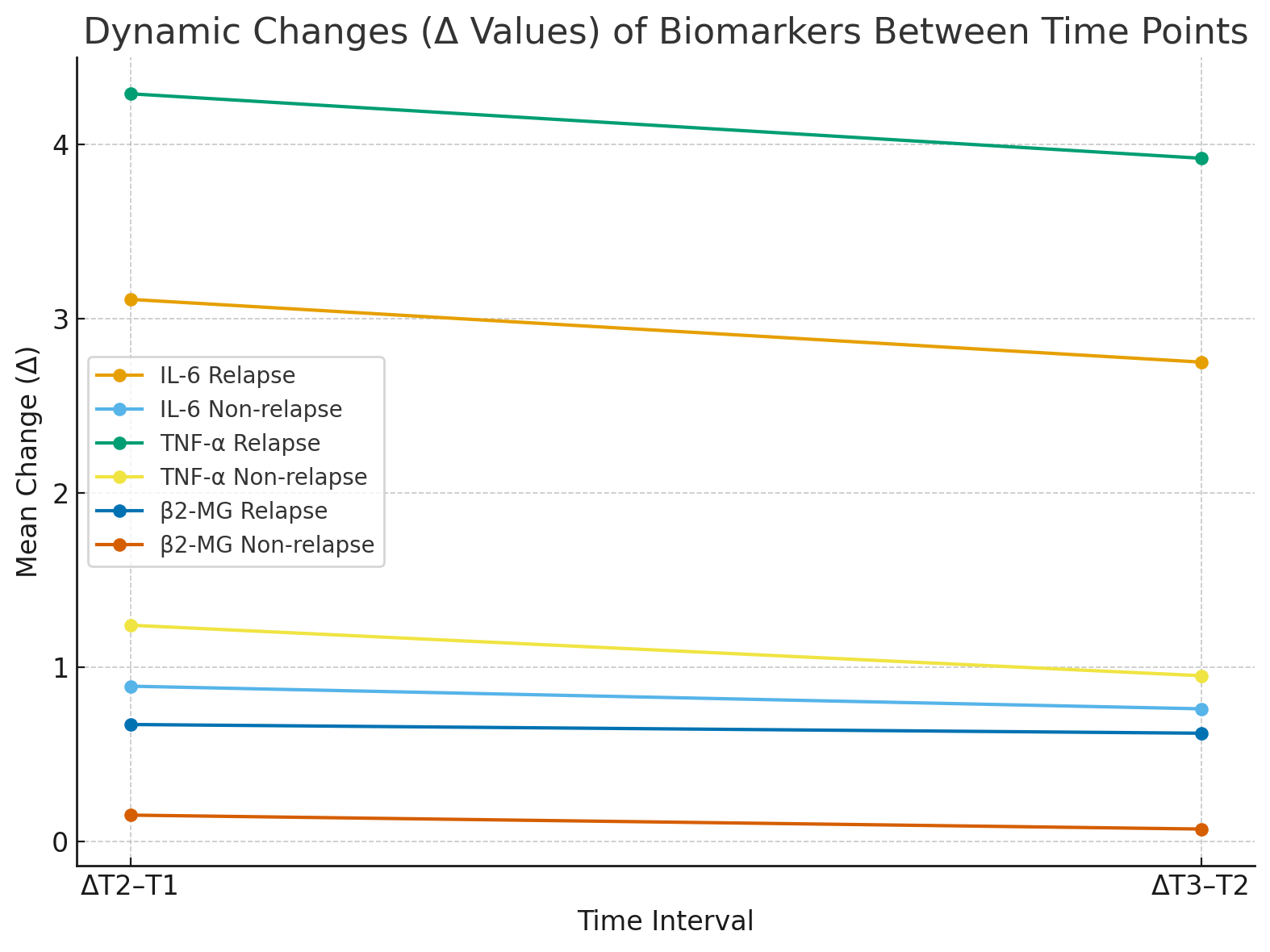


**Supplementary Figure 1. Dynamic interval changes (Δ values) of IL-6, TNF-α, and β₂-microglobulin between follow-up time points.**

Mean interval changes (ΔT2–T1 and ΔT3–T2) in serum IL-6, TNF-α, and β₂-microglobulin levels in patients with and without relapse. The relapse group exhibited consistently larger increases across both intervals, indicating early and progressively accelerating inflammatory and tumor-burden activity preceding clinical relapse. Values represent mean Δ levels for each biomarker.

**
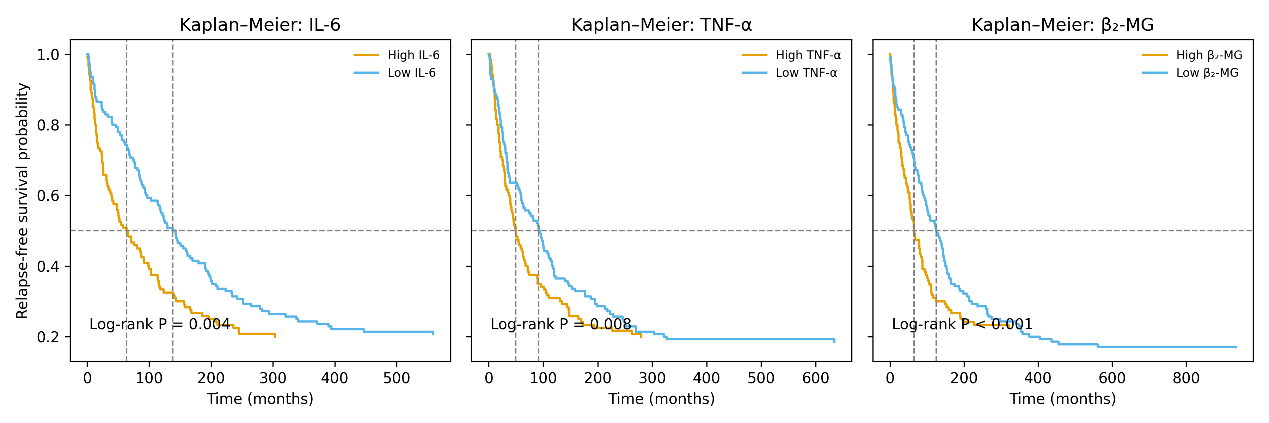
**

**Supplementary Figure 2.** Kaplan–Meier curves for relapse-free survival stratified by IL-6 (A), TNF-α (B), and β₂-microglobulin (C) levels at T2. In all panels, patients in the high biomarker group show a visibly steeper decline in relapse-free survival compared with those in the low group (all log-rank P < 0.01).


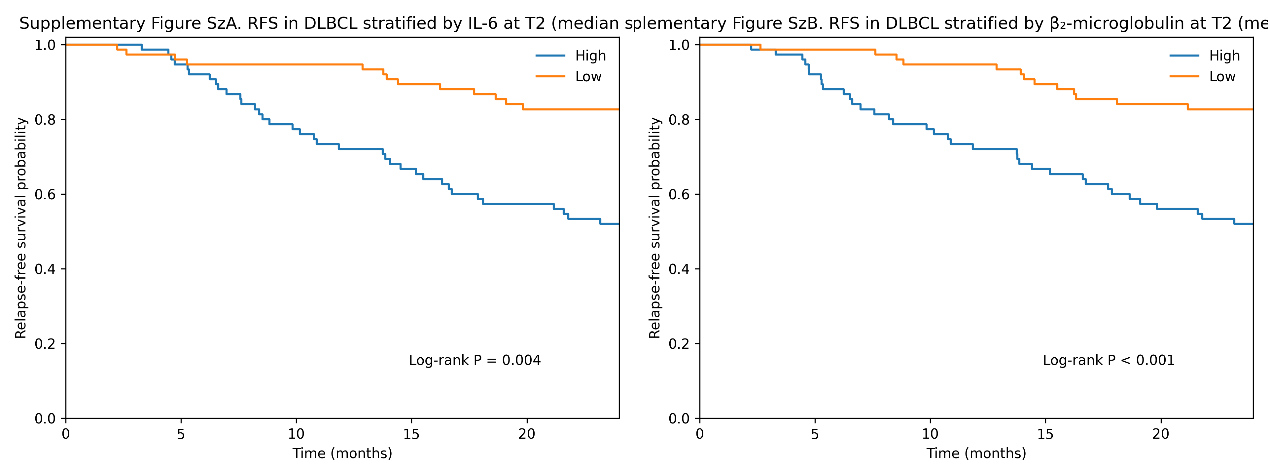


**Supplementary Figure S3**

**Kaplan–Meier curves for relapse-free survival in patients with diffuse large B-cell lymphoma (DLBCL) stratified by biomarker levels at T2.**
**(A)** Relapse-free survival stratified by serum IL-6 levels at the 3-month post-treatment time point (T2), dichotomized by the median value (log-rank *P* = 0.004).
**(B)** Relapse-free survival stratified by serum β₂-microglobulin levels at T2, dichotomized by the median value (log-rank *P* < 0.001).

**Figure note:** High and low biomarker groups were defined using the median values at T2. Relapse-free survival was estimated using the Kaplan–Meier method, and differences between groups were assessed using the log-rank test.
